# Development of a Work-Related Quality of Life Questionnaire for Medical Doctors (WQMD-9) in Japan: Questionnaire Design and Quantitative Survey

**DOI:** 10.3390/jmahp13030041

**Published:** 2025-08-19

**Authors:** Miyuki Ezura, Katsuhiko Sawada, Yusuke Takushima, Lida Teng, Ataru Igarashi

**Affiliations:** 1 Department of Corporate Planning, Otsuka Holdings Co., Ltd., Tokyo 108-8042, Japan; 2 Department of Health Technology Assessment Program, Graduate School of Health Management, Keio University, Tokyo 160-8582, Japan; 3 Clinical Development Department, Otsuka Medical Devices Co., Ltd., Tokyo 108-0075, Japan; sawada.katsuhiko@otsuka.jp; 4 Public and Government Affairs Department, Taiho Pharmaceutical Co., Ltd., Tokyo 101-8444, Japan; y-takushima@taiho.co.jp; 5 Department of Health Economics and Outcomes Research, Graduate School of Pharmaceutical Science, The University of Tokyo, Tokyo 113-0033, Japan; lidateng@gmail.com (L.T.); atarui1@mac.com (A.I.)

**Keywords:** work-related, QOL, questionnaire, medical doctors, quality of life

## Abstract

**Background**: With the ongoing development of game-changing technologies, assessing healthcare provider burden is desirable. This requires developing and evaluating subjective outcome measures, but there is no single scale that measures this burden. We developed a measure of quality of life (QOL) to address this, focusing on medical doctors (MDs). **Methods**: Based on Japan’s national statistical distribution of MDs in Japan, we qualitatively interviewed twenty MDs to identify factors that influenced their QOL and another eight MDs to verify the appropriateness and interpretability of the questions. Validity and reliability were evaluated and verified in a quantitative survey of 374 MDs to finalize the questionnaire. **Results**: Based on our initial research and interviews, we derived nine dimensions and developed the work-related QOL questionnaire for MDs (WQMD-9) accordingly. Correlation coefficients between questionnaire items were 0.3–0.7 and Cronbach’s α was 0.897, confirming the validity and reliability of the questionnaire. **Conclusions**: The WQMD-9 is an original profile-type scale with nine dimensions and five levels. We expect that as new technologies develop, evaluations of the associated medical treatment will involve measuring the QOL of not only patients but also MDs, and the WQMD-9 will facilitate this process.

## 1. Introduction

The COVID-19 pandemic forced the medical field to address the matter of healthcare provider burden. In Japan, for example, a survey conducted by the Office of Pharmaceutical Industry Research found that the percentage of respondents citing the burden on healthcare professionals as a key concern was higher than that of “burden on families” or “labor productivity” [1]. In parallel with these issues, reforms aimed at improving Japanese physicians’ working conditions took effect in April 2024.

In Japan, emergent medical technologies include drugs and medical devices, such as a mobile application for smoking cessation treatment (approved in 2020), an advanced surgical support robot [2] (approved in 2022), and the development of biopharmaceuticals and regenerative medicine technologies from low-molecular-weight compounds [3]. These new technologies have overturned conventional concepts of healthcare, and their value is related to the quality of life (QOL) of not only patients but also healthcare providers, especially medical doctors (MDs) who are directly involved with these new technologies and patients.

Reducing the burden on healthcare providers improves their QOL and is thus an aspect of the value of new medical technologies, and assessing the value of new technologies therefore requires objective evaluation of the QOL of healthcare providers. A previous systematic review [4] found that “psychology,” “work,” “QOL,” and “satisfaction” were important components for measuring MDs’ work-related QOL. However, multiple questionnaires, such as the Maslach Burnout Inventory [5], the ProQOL [6], and the SF-12 [7], and the 17-item career satisfaction instrument developed by Lepnurm et al. [8], were used, and no single pre-existing scale measured using an independent questionnaire was found. Further, in addition to evaluating healthcare provider burden from the viewpoint of resource use, such as the duration of treatment and the length of stay, the development of subjective outcome measures is necessary. Moreover, emerging concepts such as job embeddedness [9], which describes the extent to which employees are psychologically and socially attached to their workplace, should also be considered. This concept has been shown to influence healthcare professionals, including nurses, in terms of retention and innovative behavior [10]. In this study, we defined MDs’ work-related QOL as the impact of work-related factors on their subjective life satisfaction and life outside of work, and we considered the factors and content related to their work (e.g., clinical practice) that impact their work-related QOL. We developed a questionnaire that measures the QOL of MDs, the Work-related Quality of Life Questionnaire for MDs (WQMD-9), in so doing constructing an original measuring tool in accordance with standardized methods for developing a QOL profile questionnaire.

## 2. Materials and Methods

We developed the questionnaire by referring to standardized methods for developing QOL scales [11] and the COSMIN Study Design checklist for patient-reported outcome measurement instruments [12], and by conducting a survey of MDs registered in the survey company’s private database, selected according to Japan’s national statistical distribution database [13]. The survey was conducted to identify and verify content as part of the process of designing our questionnaire, rather than to collect information on the behavior and awareness of individuals. Therefore, ethical approval was not required, according to the relevant ethics committee. Participants provided their informed consent after we had explained that participation was voluntary, that the results of the investigation would not be used for other purposes, and that all responses would be fully anonymized.

We collected factors affecting MDs’ work-related QOL and prepared the first draft of the questionnaire, which had hypothetical constructs as components, accordingly. The interviews were conducted in March 2020 with 20 MDs. The participants were selected to match the national distribution of MDs in Japan: 65% hospital-based and 35% private practitioners; 50% in internal medicine, 30% in surgery, and 20% in other specialties; and 80% male and 20% female. An experienced interviewer conducted the interviews with one participant at a time, following the interview guide, and interviews lasted approximately one hour. The interview guide was prepared to collect background factors such as years of experience, managerial level, and number of staff in the department and any relevant teams, as well as the factors hypothesized to affect QOL (patients, collegial relationships, relationship with the hospital, new techniques, medical fees, and working environment and working conditions), which were asked via aided recall, and any new factors raised by the respondent via unaided recall.

The interviews were recorded and transcribed; text mining was then carried out using KH Coder (Professor Koichi Higuchi, Ritsumeikan University, Kyoto, Japan; https://khcoder.net/en/, accessed on 30 May 2025) and mind map analysis was carried out using the XMind 8 mind mapping tool (XMind, Hong Kong, China; https://xmind.app/, accessed on 30 May 2025). The elements shown by the analysis to influence work-related QOL were classified and used to construct the questionnaire. The content validity of the validated questionnaire was examined using the matrix method, and an experienced interviewer conducted interviews of approximately one hour each with eight additional MDs in October 2020 to further verify the adequacy of expressions, interpretability, and choice of response format, and to adjust the length of the entire questionnaire. The interview content was recorded and transcribed and the draft questionnaire was prepared to examine the appropriateness of expressions and wording, understandability of questions, preferred answer format, content validity, and validity of answer selection. Based on the two sets of interviews, we identified nine dimensions (workload, working time, collaboration, clinical practice, working conditions, working environment, feelings of fatigue, work–life balance, and career) and developed the questionnaire such that it had one item per dimension.

An internet survey using the draft questionnaire was carried out with 374 MDs who were recruited according to the same selection criteria based on Japan’s nationwide distribution of MDs that were used for the initial sample of 20 (65% hospital-based, 35% private; 50% internal medicine, 30% surgery, 20% other specialties; 80% male, 20% female) [13]. Participants logged in using a password and responded to the survey on a securely managed internet site dedicated to the study. The answers were collected electronically and compiled in a Microsoft Excel file. Data analysis was then performed using the JMP Version 16 statistical software (JMP Statistical Discovery LLC, Cary, NC, USA).

The results of the quantitative survey were examined by two external experts, the interpretation of the results was discussed among the project team and the external experts, and the questionnaire was finalized accordingly.

## 3. Results

### 3.1. Questionnaire Development

The results of the interviews with the initial 20 MDs are described below.

#### 3.1.1. Text Mining

Text mining was carried out via co-occurrence network analysis using frequently occurring words from the interview responses as an index, and the top 50 most commonly occurring words were examined. The most frequently occurring words were “QOL,” “time,” “hospital,” “patient,” “work,” “people,” “stress,” and “surgery.” There were no differences in response associated with the number of years of experience or medical department.

#### 3.1.2. Mind Map Analysis

The interview responses were categorized into positive (+) and negative (−) whether responses were provided based on unaided and aided recall, and the results of a one-way (+ or −) and two-way mind map analysis of the response are summarized in Table 1.

From the unaided recall responses, we extracted “free time” and “self-discretion” as a (+) reaction and “shift duty” and “on-call” as a (−) reaction. Taking these factors into account, “work–life balance (WLB),” “psychology,” and “time” were established as constructs.

In the aided recall responses to questions on the six hypothetical factors, the constructs were “treatment,” “time,” and “psychology” for patients; “working environment,” “infrastructure,” and “career” for working environment and working conditions; “career” and “infrastructure” for relationships with hospitals; “cooperation” and “psychology” for human relations; “time” and “cooperation” for medical fees; and “treatment” and “time” for new technologies.

#### 3.1.3. Constructs

Referring to the results from a previous systematic review [4], four items—“time,” “treatment,” “infrastructure/working environment,” and “cooperation”—were defined as causal elements for QOL, and three items—“WLB,” “career,” and “psychology”—were defined as outcome elements. These seven items were therefore established as the conceptual framework.

#### 3.1.4. Validation Questionnaire

In the preparation of the proposed seven-item questionnaire, it was difficult to use any existing questionnaires, so multiple questionnaires were independently prepared, and their item completeness and content validity were examined using the matrix method. Since the answers reflected the subjective opinions of the MDs, it was appropriate to carry out the interval measurement using a subjective scale. The response comprised the degree of sentiment for each item, so the step (level) rating method was used. Since the responses are generally presented using a five- to seven-point scale, we used a unipolar Likert scale using adjectives that measured responses that strongly endorsed a certain way of thinking on a scale ranging from zero (or few) to an extreme (emergency or maximum), depending on the characteristics of items, and a bipolar Likert scale using adjectives that measured responses in two directions from one extreme to its opposite and strengthened the opposite direction, with five-point response scales [11].

The questionnaire was made as simple and easy to understand as possible, and the answer form of both the unipolar and bipolar scales was set appropriately. The questionnaire consisted of five questions and five answers on the “time” item, two questions and two answers on the “cooperation” item, two questions and three answers on the “treatment” item, three questions and six answers on the “infrastructural/working environments” item, two questions and four answers on the “psychology” item, and one question and two answers on the “WLB” and “career” items.

### 3.2. Validation Interviews

The results of the validation interviews with eight additional MDs are described below.

#### 3.2.1. Appropriateness of Expression

When the appropriateness of each item was examined, it became clear that a double-barreled question should be confirmed on the “time” item; “working time” and “workload” should be asked separately; the term “colleagues” was too narrow in scope and was changed to “people who work around me;” and that for the “treatment” item, the expression “doing well” could be misunderstood to refer to the treatment outcome. For the “psychology” item, there was an opinion that “fatigue” could refer to either physical or mental (one out of eight participants) and thus was unclear, and for the “infrastructure/working environment” item, there was an opinion that it should be divided into “working conditions” and “working environment” (one out of eight participants). The “WLB” and “career” items were not considered problematic.

#### 3.2.2. Interpretability

There was an example of interpreting the “time” item as the ability to work (one out of eight participants). There was no confusion regarding the question of “time” for “intrinsic,” but in the interpretation of the “cooperation” item, two of eight participants were confused. Additionally, four of eight participants were confused about the expression “medical care and treatment” in the “treatment” question. “Fatigue” in “psychology” was interpreted as both mental and physical fatigue by six participants and mental fatigue only by the remaining two. All participants replied that the “infrastructure/working environment” item should be separated into “working conditions” and “working environment” items. There was no particular problem with the “WLB” and “career” items.

#### 3.2.3. Response Format

In examining whether there were differences in answers when the same content was asked in different ways and whether the answer differed depending on the answer format, we found that the answers differed significantly in almost all cases. The preferred answer format was bipolar for all items except the “time” and “cooperation” items, for which it was unipolar.

#### 3.2.4. Overall Questionnaire Content

Most participants expressed the opinion that the length of the questionnaire and the number of answer options (five levels) were appropriate. Participants also thought it was necessary to include the relationship with the patient, degree of cooperation of the family, and salary. The overall questionnaire content was considered appropriate.

#### 3.2.5. Drafting the Questionnaires and Preparing for Validation

Based on consultation with the experts involved in this study and considering the results of the validation interviews, we reconstructed the questionnaire with questions on nine concepts, and the answer format was set as discrete values ranging from 1 to 5. The concept of time was divided into quantity and quality: Q1 asked about “workload” and Q2 asked about the time (“working time”) that could be spent on the core work. Q3 asked about “collaboration” with colleagues (“people who work around me”). In Q4, the “treatment” item was changed to “clinical practice.” The “infrastructure/working environment” item was changed to “working conditions” (Q5) and “working environment” (Q6). The “psychology” item was explored in Q7, and “fatigue” was replaced with the phrase “feelings of fatigue,” which combines the spiritual and physical meanings. In the “WLB” item (Q8), “choice” was changed to “satisfied,” to ensure consistency with the other questions. There was no change in the “career” item (Q9).

**Figure 1:**
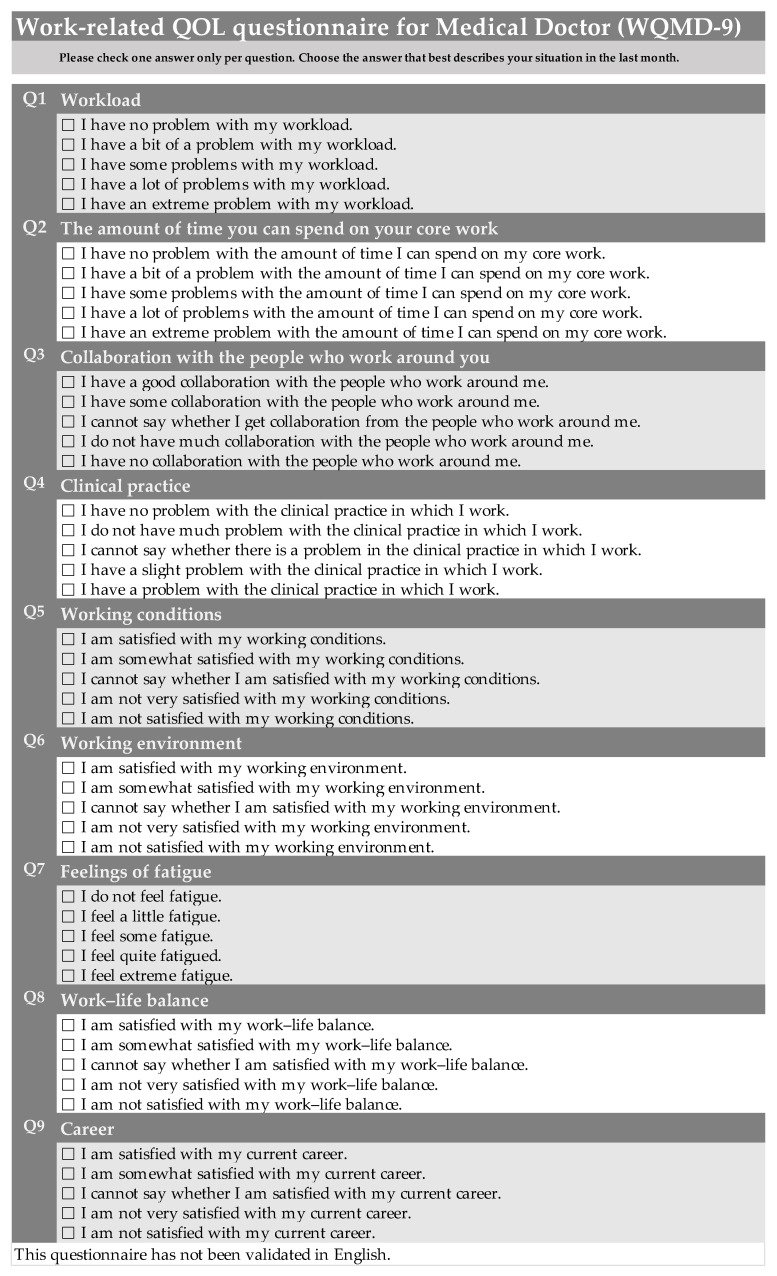
Work-related quality of life questionnaire for medical doctors (WQMD-9).

A new validation questionnaire was also prepared to confirm the content validity and was combined with the questionnaire. Q10 addressed the ideal number of patients, and Q11 addressed the actual number of patients encountered during normal working hours. Q12 was a text-based question about core working time, and Q13 and Q14 determined whether there was a difference between the ideal amount of core working time and the actual time spent doing core work, respectively. Q15 addressed the necessity of cooperation and Q16 ascertained the presence or absence of cooperation. Q17 asked whether the respondent was able to practice as they would consider ideal in their clinical practice. Q18 asked about communication with patients, which is also an important factor in clinical practice. Q19 addressed the ideal number of working hours, Q20 the actual number of working hours, and Q21 the level of satisfaction with salary and treatment. Q22 addressed the keywords extracted from the interviews on the working environment, while Q23 asked whether the necessary medical equipment and medications were available. The “psychology” item was changed to describe a more accurate condition, such as the number of days of mental fatigue (Q24) and physical fatigue (Q25) during the week. Q26 asked whether time outside of work was considered important, and Q27 addressed the “WLB” item for hours other than work. In Q28, the “career” item was revised to inquire whether it was close to ideal. The questionnaire was developed based on these considerations is shown in Figure 1.

### 3.3. Quantitative Survey

#### 3.3.1. Response Results

**Figure 2:**
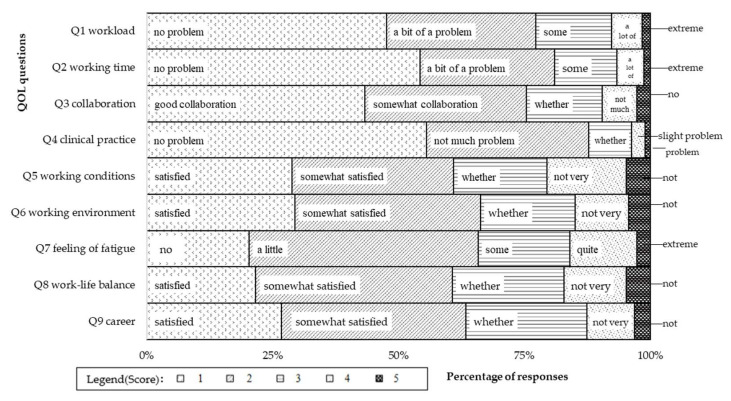
Distribution of response.

A draft questionnaire was administered to 374 MDs in March 2021. A mosaic plot of the percentage of responses to each question based on the survey was created. The questionnaire was used only to compare groups or to evaluate changes over time. The answers are scored on an ordinal scale (1 to 5), the scores are summed, and the frequency of each score is shown in Figure 2. Response scores of 1 or 2 were generally frequent and scores of 4 and 5 tended to be infrequent. This tendency was particularly marked in the responses to Q4, “clinical practice.”

#### 3.3.2. Validity

1. Construct validity.

Spearman’s correlations between the responses to Q1–Q9 and those to the validation questionnaire, i.e., Q10–Q28, to confirm their validity, are shown in Table 2. The correlation coefficients in the shaded parts of the table are the reference values for the validity assessment. Correlation values for Q2 and Q4 were lower than for other questions (0.157 for Q2 with Q13/14 and 0.276 for Q4 with Q18, respectively). However, Q2 showed a stronger correlation with other work-time-related validation questions (0.391 with Q19/20 and 0.433 with Q27) and Q4 showed a stronger correlation with the other clinical-practice-related validation question (0.422 with Q17).

2. Correlations between responses to the QOL questionnaires.

The highest correlation coefficient was between Q1 and Q2 (0.699) and the lowest correlation coefficients were between Q9 and Q2 (0.332) and Q9 and Q3 (0.328). Between-item correlation coefficients ranged from 0.3 to 0.7 [15,16].

3. Reliability.

Cronbach’s α, calculated from the questionnaire responses to Q1–Q9, was 0.897 [17,18]. To confirm the necessity of each question, we assessed the change in Cronbach’s α when one of the first nine questions was excluded from the calculation. In these cases, Cronbach’s α ranged from 0.878 to 0.897, meaning that there was no marked change in the coefficient (Table 3).

4. Factor analysis [19].

We performed exploratory factor analysis of the responses to identify potential explanatory factors. Factor loadings for two or three factors were estimated using maximum likelihood and Promax oblique rotation. In the three-factor analysis, only Q4 (“clinical practice”) was allocated primarily to the third factor (factor loading 0.952). Further, the answers to Q4 exhibited greater heterogeneity than those to the other questions. We therefore excluded Q4 and performed two-factor analysis. In this analysis, Q1 (“workload,” factor loading 0.911), Q2 (“working time,” 0.675), Q5 (“working conditions,” 0.697), Q6 (“working environment,” 0.770), and Q9 (“career,” 0.695) could be decomposed into factors with higher loadings (Table 4).

### 3.4. Finalizing the Questionnaire

The results of the quantitative survey were examined by external experts because the responses to Q4 (“clinical practice”) showed a tendency to exhibit a ceiling effect and the exploratory factor analyses suggested heterogeneity with the other questions in the answers. We then solicited the opinion of four other MDs about the interpretation of “clinical practice” and about the answer format (unipolar or bipolar). This revealed (1) that clinical practice is understood as a broader concept that includes activities such as treatment and examination; (2) that using a bipolar format makes the questions easier to answer as it allows for flat answers; and (3) that the questions are easier to answer when the timeframe is specified. Consequently, the use of the term “clinical practice” was not considered problematic; however, the fact that the answer format was unipolar (no, slight, moderate, severe, unable in an earlier version of the WQMD-9) was considered a potential cause of the ceiling effect, and it was changed to a bipolar (no problem, not much problem, cannot say, slight problem, or have a problem in WQMD-9) answer format. Assuming that this was the cause of the ceiling effect, we finalized the questionnaire. The study’s experts indicated that it was better to limit the timeframe covered by the questionnaire as a whole and to specify a timeframe of “the previous month,” based on the review period (the previous month) in the Medical Outcomes Study (MOS) 36-Item Short Form Health Survey (SF-36) QOL profile measuring tool [20]. Our questionnaire, the WQMD-9, was then examined as a measurement tool (Figure 1). Nonetheless, the source version of the questionnaire was composed in Japanese.

## 4. Discussion

In the first stage of the study, items were identified based on qualitative interviews that sought to identify items as relevant components of an MD’s work-related QOL. A systematic literature review showed that four components—“psychology-related,” “work-related,” “satisfaction-related,” and “QOL-related”—should be used when conducting work-related QOL surveys [4]. “QOL” was also frequently encountered during text mining, but we removed it from the construct because it was directly connected to the study goal. “Satisfaction” is a unique concept that deviates from the concept of QOL, and we decided it should refer to “career” because it is important to clearly specify what type of satisfaction is intended in a questionnaire [21].

When preparing the validation questionnaire, when we analyzed the item completeness and content validity of the questionnaire content, we found that “work environment,” “treatment,” and “working time” were highly correlated with other questions. “Salary” and “patient” were highly independent, but they were summarized in “infrastructure” and “treatment,” respectively. In the interviews with the eight additional MDs using the validation questionnaire, the opinion that “relationship with the patient” and “salary” were essential to the whole questionnaire was expressed by one participant for each item. Since these were factors with high independence in the first assessment stage, we could interpret this as support. However, after discussions with our study’s experts on the inclusion of highly independent questions and comprehensive questions, we concluded that it would be acceptable to include the patient relationship in “treatment” and remuneration (salary) in “working conditions”. Additionally, in the quantitative survey, we found that the correlation coefficient between “clinical practice” and the verification question of the “patient relationship” was 0.276, and the correlation coefficient between “working conditions” and the verification question of “salary” was 0.569, so including salary as a separate item had little effect on the findings.

Considering the low correlation coefficient of 0.157 for the answers to Q2, “working time,” and the applicable verification questions (“How many hours a day do you have for your core work at present?/How many hours a day is the ideal time for your core work?”) in the quantitative survey, we determined that the problems with recognizing “core work” were not limited to the timescale. The “core work” wording was accurately interpreted in approximately 80% of the descriptive responses, so we concluded that there were no problems with the interpretability of that question.

In the responses to Q4, “clinical practice,” we observed a ceiling effect. The percentages of response scores of 4 and 5 were also low in Q1, “workload”, and Q2, “working time”, but this phenomenon was not ascribed to a ceiling effect because it could be balanced against other response scores. For Q4, however, its heterogeneity was confirmed in the three-factor analysis, and it seemed to be due to the ceiling effect. We interviewed four MDs about their interpretation of the wording “clinical practice” and the question and answer formats to investigate the cause of this ceiling effect. Consequently, we discussed with external experts and revised in the final questionnaire, using the bipolar answer format. However, further validation is required.

The quantitative study involving 374 MDs using the pre-WQMD-9 showed that the correlation coefficients were within the range of 0.3–0.7 for correlations among answers to the questions and between the questions in the final and validation questionnaires [15,16], and the construct validity of the questionnaire was thus verified. Cronbach’s α was 0.897 [17,18], and the reliability was therefore assumed to be assured.

This study has some limitations. First, it began with the development of a questionnaire to measure the work-related QOL of healthcare professionals. However, we only surveyed MDs because the definition of healthcare professional differed across countries in a previous systematic review [4]. Second, we examined the WQMD-9 in this study and will only verify its validation and reliability in the next stage of this research. Finally, this work entailed exploratory factor analysis only, and did not yet involve validation of the factors for the WQMD-9 questionnaire. Future work using confirmatory factor analysis may therefore be required to generate conclusive findings. Therefore, a limitation of this study is that it lacks a validation report for the WQMD-9.

## 5. Conclusions

In this study, we provide a detailed description of the process of developing a questionnaire measuring work-related QOL in MDs, which we did using qualitative and quantitative approaches. The WQMD-9 questionnaire clarifies the definition [22] of MDs’ work-related QOL. This work provides preliminary verification of the internal validity of the WQMD-9; however, further validation and refinement are necessary.

## Figures and Tables

**Table 1:** Summary of mind map analysis results.

Category	Simplex (+) Factor	E *	U ^†^	Simplex (−) Factor	E *	U ^†^	Two-Way (+/−) Factor		E *	U ^†^	Construct
Unaided recall	Successful treatment and surgery	0	1	Watch on call	2	4					Work–life balance Psychology Time
	Free time	3	6	Training for residents	0	2					
	Salaries and allowances	1	2	Miscellaneous	1	1					
	Own discretion	1	0	Executive policies	1	2					
Aided recall											
Patient	Successful treatment and surgery	4	9	Rapid response and emergency	1	6	Large number of patients	+	1	3	Treatment Time Psychology
				Idle chitchat	1	2		−	1	5	
				Patients’ family response	2	5	Difficult treatment	+	0	2	
				Patient comprehension	1	12		−	0	3	
Work environment Working Conditions	Number of doctors and staff	5	2	Watch on call	3	3	Research and academic societies	+	4	8	Work environment Infrastructure Carrier
	Free time, leisure	1	3	Conference	0	5		−	0	6	
	Adequacy of facilities	1	5	Training for residents	0	1					
	Holiday	1	0								
	Commuting time	1	4								
	Surgical opportunity	0	3								
Relationship with the hospital	Institutional reputation	1	3	Executive policies	1	1	Promotion	+	0	3	Carrier Infrastructure
	Salary and allowances	2	9	Competition from other facilities	1	0		−	0	1	
	Own discretion	0	2	Relationships with other medical departments	1	0					
				Miscellaneous	2	2					
Human relations							Doctor	+	1	3	Cooperation Psychology
								−	1	12	
							Nurse	+	0	1	
								−	0	5	
							MR	+	0	3	
								−	0	2	
							Staff	+	7	10	
								−	1	2	
Medical fees							Medical fees	+	1	6	Cooperation /Time
								−	1	5	
New technology							New technology	+	3	10	Treatment /Time
								−	0	7	

* E, emphasized, i.e., responses with modifiers such as “very,” “extremely,” and “especially.” ^†^ U, usual, i.e., answers without modifiers. +, positive response. −, negative response. Note: Partially modified from reference [14].

**Table 2:** Correlation coefficients for each response and quantity index question.

Validation Questionnaire		Number of Patients	Primary Business	Cooperation Status	State of Medical Care	Patient Relationship	Working Hours	Salary	Equipment System	Equipment * Drugs	Strong Fatigue	Time Outside Work	Career
		Q10/11	Q13/14	Q15/16	Q17	Q18	Q19/20	Q21	Q22	Q23	Q24/25	Q27	Q28
		Quant ^†^	Quant ^†^	Qual	Qual	Qual	Quant ^†^	Qual	Qual	Qual	Quant ^†^	Qual	Qual
Mean (Median)		1.39(1.25)	1.16(1.14)	―	―	―	1.43(1.25)	―	―	―	3.7(3)	―	―
Q1	workload	0.322	0.213	0.135	0.346	0.181	0.407	0.355	0.275	0.109	0.540	0.478	0.278
Q2	working time	0.300	0.157	0.206	0.278	0.231	0.391	0.346	0.312	0.167	0.425	0.433	0.171
Q3	collaboration	0.157	0.094	0.306	0.380	0.137	0.143	0.231	0.318	0.214	0.316	0.285	0.276
Q4	clinical practice	0.208	0.166	0.073	0.422	0.276	0.204	0.261	0.350	0.227	0.415	0.269	0.324
Q5	working conditions	0.345	0.189	0.101	0.413	0.180	0.358	0.569	0.304	0.189	0.477	0.417	0.396
Q6	working environment	0.307	0.130	0.192	0.384	0.182	0.273	0.473	0.477	0.317	0.467	0.410	0.395
Q7	feelings of fatigue	0.321	0.179	0.110	0.394	0.205	0.332	0.385	0.318	0.180	0.661	0.426	0.299
Q8	work–life balance	0.329	0.220	0.147	0.408	0.221	0.386	0.433	0.383	0.224	0.508	0.603	0.388
Q9	career	0.207	0.075	0.105	0.404	0.153	0.272	0.452	0.355	0.271	0.327	0.299	0.661

* Correlation coefficients are Spearman (absolute). ^†^ Mean and median are quantitative only. Note: Partially modified from reference [14].

**Table 3:** Cronbach’s α between questionnaire item responses.

		Cronbach α	
Q1	workload	Q1 exclusion	0.881
Q2	working time	Q2 exclusion	0.885
Q3	collaboration	Q3 exclusion	0.897
Q4	clinical practice	Q4 exclusion	0.888
Q5	working conditions	Q5 exclusion	0.879
Q6	working environment	Q6 exclusion	0.881
Q7	feelings of fatigue	Q7 exclusion	0.884
Q8	work–life balance	Q8 exclusion	0.878
Q9	career	Q9 exclusion	0.894

**Table 4:** Factor analysis.

	Three Factors				Two Factors	
Parameters	Rotated Factor Loading			Parameters	Rotated Factor Loading	
	Factor 1	Factor 2	Factor 3		Factor 1	Factor 2
Q6 working environment	0.804	−0.020	0.051	Q6 working environment	0.770	0.280
Q8 work–life balance	0.698	0.221	−0.069	Q5 working conditions	0.697	0.420
Q5 working conditions	0.662	0.167	0.036	Q9 career	0.695	0.400
Q9 career	0.628	−0.034	0.041	Q8 work–life balance	0.594	0.203
Q3 collaboration	0.330	0.095	0.196	Q3 collaboration	0.423	0.287
Q1 workload	−0.040	0.979	0.008	Q1 workload	0.297	0.911
Q2 working time	0.128	0.644	0.060	Q2 working time	0.364	0.675
Q7 feeling of fatigue	0.360	0.393	0.066	Q7 feeling of fatigue	0.498	0.522
Q4 clinical practice	0.040	0.047	0.952			

## Data Availability

Raw data were generated at Intage Healthcare Inc. Analyzed data supporting the findings of this study are available from the corresponding author upon reasonable request.

## Abbreviations

The following abbreviations are used in this manuscript:

QOL	Quality of Life
MDs	Medical Doctors
WQMD-9	the Work-related Quality of Life Questionnaire for Medical Doctors
COSMIN	Consensus-based Standards for the selection of health Measurement Instruments
WLB	Work–Life Balance
MOS	the Medical Outcomes Study
SF-36	36-Item Short-Form Health Survey
